# Suppression of Human Liver Cancer Cell Migration and Invasion via the GABA_A_ Receptor

**DOI:** 10.3969/j.issn.2095-3941.2012.02.002

**Published:** 2012-06

**Authors:** Zhi-ao Chen, Mei-yan Bao, Yong-fen Xu, Ruo-peng Zha, Hai-bing Shi, Tao-yang Chen, Xiang-huo He

**Affiliations:** 1 State Key Laboratory of Oncogenes and Related Genes, Shanghai Cancer Institute, Renji Hospital, Shanghai Jiao Tong University School of Medicine, Shanghai 200032, China; 2 Qidong Liver Cancer Institute, Qidong 226200, China

**Keywords:** γ-aminobutyric acid (GABA), receptor, migration, invasion, hepatocellular carcinoma

## Abstract

**Objective:**

To investigate the roles of the γ-aminobutyric acid (GABA) in the metastasis of hepatocellular carcinoma (HCC) and to explore the potential of a novel therapeutic approach for the treatment of HCC.

**Methods:**

The expression levels of GABA receptor subunit genes in various HCC cell lines and patients‘ tissues were detected by quantitative real-time polymerase chain reaction and Western blot analysis. Transwell cell migration and invasion assays were carried out for functional analysis. The effects of GABA on liver cancer cell cytoskeletal were determined by immunofluorescence staining. And the effects of GABA on HCC metastasis in nude mice were evaluated using an *in vivo* orthotopic model of liver cancer.

**Results:**

The mRNA level of GABA receptor subunits varied between the primary hepatocellular carcinoma tissue and the adjacent non-tumor liver tissue. GABA inhibited human liver cancer cell migration and invasion via the ionotropic GABA_A_ receptor as a result of the induction of liver cancer cell cytoskeletal reorganization. Pretreatment with GABA also significantly reduced intrahepatic liver metastasis and primary tumor formation *in vivo*.

**Conclusions:**

These findings introduce a potential and novel therapeutic approach for the treatment of cancer patients based on the modulation of the GABAergic system.

## Introduction

Hepatocellular carcinoma (HCC) is one of the most common lethal cancers in the world ^[^^1^^]^. In endemic areas, HCC prevalence is approximately 150 cases per 100,000 people. Although numerous clinical achievements were realized in HCC during the past few decades, the five-year survival of liver cancer patients remains relatively low at a rate of only 6% in the United States ^[^^2^^-^^4^^]^. The majority of deaths associated with HCC are due to the metastasis of original tumor cells^[^^5^^]^. Thus, the major focus of tumor biological research is the factors that regulate tumor cell migration and invasion.

Neurotransmitters are important initiators of migratory activity ^[^^6^^]^. Our previous study, which was based on large-scale cDNA transfection screening, showed that neurotransmitter-related genes such as γ-aminobutyric acid (GABA) and receptor-associated protein are involved in HCC cell proliferation or survival ^[^^7^^]^. Thus, the theory that the GABAergic system is involved in HCC progression is supported. GABA reportedly contributes to the proliferation, differentiation, and migration of several kinds of cells, including cancer cells, in addition to its inhibitory neurotransmitter role in the nervous system ^[^^8^^]^. Masaharu et al. were the first group to establish a possible relationship between cancer and GABA ^[^^9^^]^. This same group subsequently showed that GABA, with the action of metabotropic receptors, attenuates azoxymethane-induced carcinogenesis in the rat colon ^[^^10^^]^. However, controversial reports were documented regarding the positive or negative regulation of GABA in cancer tumorigenesis ^[^^11^^-^^20^^]^. GABA is a principal inhibitory neurotransmitter that is predominantly synthesized from glutamate ^[^^21^^]^. GABA and its associated receptors can have varied effects depending on the tumor origin because GABA mediates these effects by the activation of traditional ionotropic (GABA_A_ or GABA_C_) and metabotropic (GABA_B_) receptors ^[^^22^^]^. More importantly, these studies highlight the complexity of the GABA receptor pathway.

The mammalian liver has a sodium-dependent, bicuculline-insensitive GABA transport system and a sodium-independent, bicuculline-sensitive GABA_A_ receptor system^[^^23^^]^. For the role of the GABAergic system in HCC, Munik et al.^[^^24^^]^ demonstrated that increased GABA_A_ receptor activity inhibits the expression of α-fetoprotein mRNA and the proliferation of the HepG2 human hepatoblastoma cell line. Subsequently, they found that GABA_A_ β3 receptor expression is down-regulated in human HCC while the restoration of GABA_A_ β3 receptor expression results in attenuated tumor growth in nude mice ^[^^25^^]^. Baclofen, a GABA_B_ receptor agonist, inhibits human HCC cell growth *in vitro* and *in vivo* ^[^^26^^]^. Thus, malignant hepatocytes are deprived of or have low GABAergic activity, which suggests that these abnormalities of the GABAergic system may contribute to the pathogenesis of hepatic carcinogenesis or HCC cell proliferation. However, the precise role of GABA and its receptors in human liver cancer cell migration and invasion is not well understood.

This study investigated the roles of the GABAergic system in the metastasis of HCC and explored the potential of a novel therapeutic approach for the treatment of HCC.

## Materials and Methods

### Human tissues

Human primary HCC (*n*=50) and adjacent non-tumor (NT) liver tissues (3 cm from the tumor) were collected from surgical specimen archives of the Qidong Liver Cancer Institute, Jiangsu Province, China. The study protocol was approved by the Ethical Review Committee of the World Health Organization Collaborating Center for Research in Human Production, as authorized by the Shanghai Municipal Government. All of the patients signed a written informed consent.

### Cell culture and reagents

SK-Hep1 cells (ATCC, ATCC No. HTB52) were maintained in Dulbecco’s modified Eagle medium (DMEM) (Invitrogen, Carlsbad, CA), and were supplemented with 10% fetal bovine serum (Invitrogen), 100 U/mL penicillin G, and 100 mg/mL streptomycin sulfate (Sigma-Aldrich, St. Louis, MO). SMMC-7721 cells purchased from the Shanghai Second Military Medical University were cultured in DMEM with 10% newborn calf serum (Invitrogen). All cells were cultured at 37°C with 5% CO_2_.

GABA, gaboxadol hydrochloride (T101, GABA_A_ receptor agonist, 10 µM), muscimol hydrobromide (GABA_A_ receptor agonist, 10 µM), 1(S), 9(R)-(−)-bicuculline methbromide (B7561, GABA_A_ receptor antagonist, 10 µM), SR-95531 (S106, GABA_A_ receptor antagonist, 10 µM), CGP 35348 hydrate (C5851, GABA_B_ receptor antagonist, 10 µM), and 2-hydroxysaclofenwere (A6566, GABA_B_ receptor antagonist, 10 µM) were obtained from Sigma-Aldrich and dissolved in H_2_O. (±)-Baclofen (GABA_B_ receptor agonist, 10 µM) (Sigma-Aldrich) was dissolved in dimethyl sulfoxide (DMSO)/1 M HCl.

### RNA extraction and quantitative real-time polymerase chain reaction

Total RNA was extracted from tissues or cells using the TRIzol reagent (Invitrogen) according to the manufacturer’s instructions. Reverse-transcription (RT) polymerase chain reactions (PCR) were carried out with the PrimeScript RT reagent Kit (TaKaRa, Dalian, China). The expression levels of GABA receptor subunit genes were determined by quantitative real-time PCR and were normalized against an endogenous β-actin control using SYBR Premix Ex Taq (TaKaRa). Data were analyzed using a ΔΔCt approach and were expressed as the target gene/β-actin ratio [2^-ΔCt(target gene-β-actin)^]. The primers for real-time PCR are shown in **Table 1**.

**Table 1:** Primer sequence used for real-time PCR.

Oligo name	Accession code	Sequence (5′-3′)	Expected length (bp)
AR α1-for	NM_000806	AGAAAAACAACACTTACGCTCCA	119
AR α1-rev		GGGCTTGACCTCTTTAGGTTC	
AR α2-for	NM_000807	AGTGGCTGTTGCCAATTATGC	249
AR α2-rev		GGACTGACCCCTAATACAGGTT	
AR α3-for	NM_000808	CATGAAGATCCTTCCACTGAACA	139
AR α3-rev		GGTTCCGTTGTCCACCAATC	
AR α4-for	NM_000809	CCCCAGGACAGAACCAAAAGG	120
AR α4-rev		CTGTAACAGGACCCCCAAATC	
AR α5-for	NM_000810	CATCGCTCACAACATGACCAC	125
AR α5-rev		CCATCGGGAAGTCCTCAAGC	
AR α6-for	NM_000811	ATTCTGTGGCTAGAAAATGCCC	115
AR α6-rev		GCCGCAGCCGATTGTCATA	
AR β1-for	NM_000812	AAGGATATGACATTCGCTTGCG	134
AR β1-rev		CTGCTGGAAATACATGGTGAGT	
AR β2.1-for	NM_021911	CCCTTCTGGAATATGCCCTAGT	205
AR β2.1-rev		CGTCTAGTTGGGGAGAGGTTTC	
AR β2.2-for	NM_000813	GCAGAGTGTCAATGACCCTAGT	137
AR β2.2-rev		TGGCAATGTCAATGTTCATCCC	
AR β3-for	NM_021912	CAAGCTGTTGAAAGGCTACGA	84
AR β3-rev		GCGATGTCGATGTTCATCCC	
AR γ1-for	NM_173536	TTCTGCGGAGTCAAAGTAGAGG	60
AR γ1-rev		CCAAATGCAGGGTCAGTAACAA	
AR γ2-for	NM_000816	GAAGCTCAGTCTACTCGACTCC	141
AR γ2-rev		AGACCCATGTTTTGTTAGAAGCA	
AR γ3-for	NM_033223	ACTCCTGCCCGCTGATTTTC	233
AR γ3-rev		TGTCTGAATGGTGAAGTATCCCA	
AR θ-for	NM_018558	TCCCGAAATTCCACTTCGAGT	240
AR θ-rev		ACATCGTGATCGTGTAGTCCA	
AR π-for	NM_014211	ACTTGGCCTTCGTGTGTCTG	176
AR π-rev		CGCTATCTGTACGGGTTCTCC	
AR δ-for	NM_000815	TCGACCACATCTCAGAGGC	168
AR δ-rev		ACTTGGCGTTCACGATGAAGG	
AR ε2-for	NM_021987	TCTCACTCTTGCCCTCTATCTTT	210
AR ε2-rev		CCTGCTCACATTGAAGAAAATCG	
BR1.1-for	NM_001470	CCAACGCCACCTCAGAAGG	236
BR1.1-rev		GGAGCAGATTCGGACACAGC	
BR1.2-for	NM_021903	CTGGGGCTCGATGGTTACC	288
BR1.2-rev		GGCAAATGTCTCAATGGTCCG	
BR1.3-for	NM_021904	AACGCCACACTCAGAACGG	184
BR1.3-rev		TGGATCACACTTGCTGTCGTG	
BR1.4-for	NM_021905	CGCTGTGTCCGAATCTGCT	117
BR1.4-rev		GGGGTCACACCGGAAATCC	
BR2-for	NM_005458	CCGCAACGAGTCACTCCTG	102
BR2-rev		TTTATCGCATCGTAGAAGGCTTT	

Notes: AR, GABA_A_ receptor, BR, GABA_B_ receptor.

### Migration and invasion assays

Cells were placed in their growth media in six-well plates at 4 × 10^5^ cells/well until 65% to 70% confluence was achieved. The medium was replaced by DMEM, without serum and supplements, after three washes with PBS and was incubated for 24 h. The cells were incubated with increasing concentrations of 1 µM-10 µM GABA and GABA_A_ or GABA_B_ receptor agonists at indicated concentrations. After 24 h, the cells were trypsinized and counted. From the cells in the serum-free DMEM, 1 × 10^5^ was placed into the upper chamber of each cell culture insert (BD Bioscience, MA) with or without 150 µg matrigel (BD Biosciences). Briefly, for the migration assay, the cells were placed into the upper chamber of each insert with the non-coated membrane. For the invasion assay, the cells were placed on the upper chamber of each insert coated with 150 µg matrigel. In both assays, 800 µL of the medium supplemented with 10% serum was injected into the lower chambers. After several hours (4 h for SK-Hep-1 and 12 h for SMMC-7721 in migration assays, and 17 h for SK-Hep-1 and 31 h for SMMC-7721 in invasion assays) of incubation at 37°C, the cells that were transferred to the lower membrane of the inserts and were fixed and stained using a dye solution that contained 0.1% crystal violet and 20% methanol. The cells were then imaged in five fields for each membrane and counted using an IX71 inverted microscope (Olympus Corp, Tokyo, Japan).

To identify a potential target receptor, cells were seeded in 6-well plates that were deprived of serum and supplements for 24 h. GABA was then added to the culture medium with or without pre-incubation with GABA_A_ or GABA_B_ receptor antagonists for 2 h. The metastasis of cancer cells is facilitated by their ability to migrate as described above.

### Western blot analysis

Cells treated with different stimulations were harvested by scraping into a SDS sample buffer that contained a cocktail of protease inhibitors and PhosSTOP Phosphatase Inhibitor (Roche, Pleasanton, CA). Similar amounts of proteins were loaded into the gel, separated by 8% to 12% SDS-PAGE gel electrophoresis, and transferred to a nitrocellulose membrane (Bio-Rad, Hercules, CA). The membrane was blocked with TBST (0.05% Tween 20 in TBS) that contained 5% skim milk, and then incubated overnight with rabbit anti-GABRA3 and mouse anti-GABBR1 (Santa Cruz, Santa Cruz, CA) (1:500) at 4°C. The membrane was again washed three times in TBST and subsequently incubated with an horseradish peroxidase (HRP)-conjugated secondary antibody (Pierce, Rockford, IL) (1:2000) for 2 h at room temperature. The membranes were stripped of their primary antibodies and reprobed with antibodies as necessary. The immunocomplexes were detected using enhanced chemiluminescence (Pierce, Rockford, IL).

### Immunofluorescence staining

After pre-incubation with 10 µM GABA for 24 h, the cells were washed with PBS and then fixed with 3.4% paraformaldehyde in PBS for 10 min. The cells were permeabilized with 0.1% Triton X-100 in PBS for 10 min at room temperature and incubated in blocking buffer supplemented with 1% FBS for 1 h at room temperature. The cells were incubated overnight with Alexa Fluor^®^ 594 phalloidin (Invitrogen) and with a α-tubulin mouse monoclonal antibody (1:200 dilution, Sigma) at 4°C. The cells were washed three times in PBS and then stained with Alexa Fluor^®^ 488 Goat Anti-Mouse IgG (1:200 dilution, Invitrogen) for 2 h at room temperature. After incubation with 4’-6-diamidine-2-phenyl indole (DAPI) to stain the nuclei, the cells were examined using a confocal laser scanning microscope (Olympus Corp.).

### *In vivo* metastasis assays in nude mice

For *in vivo* metastasis assays, 1 × 10^6^ SMMC-7721 cells were pretreated with 10 µM GABA or H_2_O and suspended in 40 µL serum-free DMEM/matrigel (1:1) prior to injection into each mouse. Each nude mouse (4- to 6-week-old male BALB/c-nu/nu mice, *n*=10 for each group) was anesthetized and orthotopically inoculated in the left hepatic lobe through an 8 mm transverse incision in the upper abdomen by using a micro-syringe. After 8 weeks, the mice were sacrificed, and their livers and lungs were dissected, fixed with phosphate-buffered neutral formalin, and prepared for standard histological examination. The mice were manipulated and housed according to protocol, as approved by the Shanghai Medical Experimental Animal Care Commission.

### Statistical analysis

The experiments were repeated at least three times, and the results were expressed as the mean and the standard error of the mean (SEM). Student’s *t*-tests (two-tailed) and one-way ANOVA analysis were used to compare the means of two or more samples, unless indicated otherwise. The statistical significance of GABA receptor mRNA expression levels in HCC patients was determined by non-parametric Mann-Whitney U tests for unpaired observations. The results were considered significant when *P* values were less than 0.05. All statistical analyses were performed using the SPSS V15 package.

## Results

### Expression levels of GABA receptors in HCC

**Figure 1:**
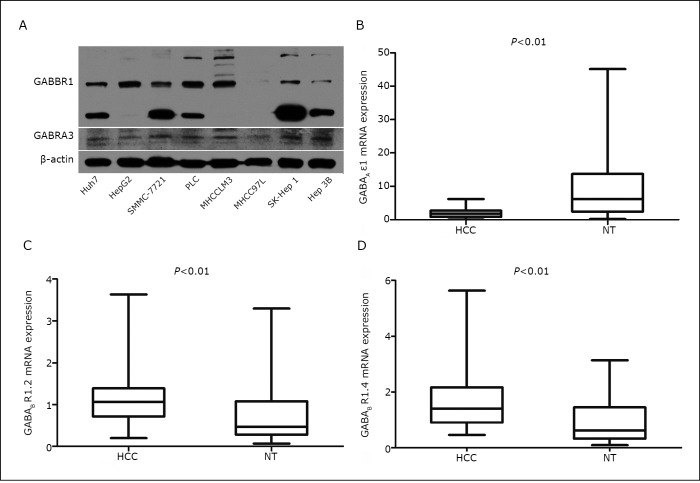
Expression levels of GABA receptors in HCC. A: Expression levels of GABA_A_ Rα3 (GABRA3) and GABA_B_ R1 (GABBR1) protein in liver cancer cells. Cell lysates were examined by Western blot analysis using equal amounts of proteins. B, C, D: The relative mRNA expressions of GABA_A_ receptor ε1 subunits, GABAB R1.2, and GABA_B_ R1.4 in HCC and in adjacent non-tumorous liver tissue samples (NT) (*n*=50) were determined by quantitative real-time PCR.

As of this writing, 16 human GABA_A_ receptor subunits (α1-6, β1-3, γ1-3, δ, ε, π, and θ) and two human GABA_B_ receptor subunits (R1 and R2) have been cloned ^[^^27^^, ^^28^^]^. To identify the patterns of GABA receptor expression in HCC, the expression of GABA receptor subunits in various HCC cell lines was examined using real-time PCR and Western blot analysis. As shown in **Figure 1A** and **Table 2**, most cell lines express the GABA_A_ receptor α3 and ε, and the GABA_B_ R1 (BR1.2 and BR1.4) subunits. Therefore, the expression levels of the GABA_A_ receptor α3 and ε, and the GABA_B_ R1.2 and R1.4 subunits was determined in primary HCC tissues as well as adjacent NT liver tissues. The results revealed that the mRNA level of the GABA_A_ receptor subunit ε1 was lower in HCC tissues than in NT liver tissues (**Figure 1B**, *n*=50), but no significant difference in the expression of the GABA_A_ receptor α3 subunit was found between the two groups (data not shown). The mRNA levels of GABA_B_ R1.2 and GABA_B_ R1.4 were higher in HCC tissues than in NT liver tissues (**Figure 1C**). These data suggest the possible involvement of the GABAergic system in HCC.

**Table 2:** Quantitative analysis of the mRNA expression of GABA receptor subunits in HCC cell lines.

	Huh7	HepG2	SMMC-7721	PLC	MHCCLM3	MHCC97L	SK-Hep1	Hep3B
AR α1	+	-	-	-	-	-	-	-
AR α2	++++	-	-	-	-	-	-	+++
AR α3	+	+	+++	+++	+	+	+	-
AR α4	+	-	-	-	-	-	+	+
AR α5	-	-	-	-	-	-	-	-
AR α6	-	-	-	-	-	-	-	-
AR β1	++	-	-	-	-	-	-	++
AR β2.1	-	-	-	-	-	-	-	-
AR β2.2	-	-	-	+	-	-	-	-
AR β3	-	-	-	-	-	-	-	-
AR γ1	-	-	-	-	-	-	-	-
AR γ2	-	-	-	+	-	-	-	-
AR γ3	-	-	-	+	-	-	-	-
AR ε2	+	+	++	-	++	++	-	-
AR δ	-	-	++	-	-	-	-	-
AR θ	-	+	-	-	-	+	+	-
AR π	-	-	+	+	-	-	-	-
BR 1.1	+	-	+	-	+	+	++	-
BR1.2	++	++	+++	++	++	++	+++	++
BR1.3	++	+	+	+	+	+	++	+
BR1.4	++	+	++	+	++	++	+++	++
BR 2	-	-	-	-	-	-	+	-

Notes: AR, GABA_A_ receptor, BR, GABA_B_ receptor. The gene is depicted as the number of transcripts per 10^3^ copies of the housekeeping gene β-actin, -, <0.01,+, ≥0.01, ++, ≥0.1, +++, ≥1, ++++, ≥10.

### GABA-inhibited HCC migration and invasion

**Figure 2:**
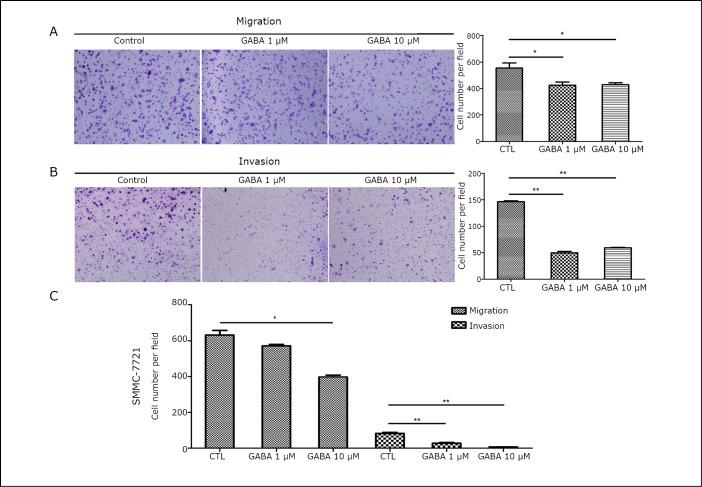
GABA-inhibited HCC migration and invasion. A, B: Migration and invasion assays were performed in SK-Hep1 cells stimulated with various concentrations of GABA (1 µM and 10 µM) for 24 h. Representative images are shown on the left, and the quantification of 5 randomly selected fields is shown on the right. C: Migration and invasion assays were performed in SMMC-7721 cells stimulated with GABA for 24 h. Data are representative of 3 independent experiments and are shown as means ± SEM. (*, *P*<0.05, **, *P*<0.01, by one-way ANOVA analysis).

To determine the effect of exogenous GABA on HCC cell metastasis, SK-Hep1 and SMMC-7721 cell lines with relatively high levels of GABA_A_ and GABA_B_ receptors (**Figure 1A** and **Table 2**) were treated with various concentrations of GABA without serum for 24 h. Cell proliferation and colony formation assays showed that GABA has no impact on liver cancer cell growth (data not shown). A filter with or without matrigel coating was used as a model for the basement membrane. The number of cells that invaded the lower compartment of the chamber through the filter with GABA stimulation was significantly lower than that in the cells without GABA (**Figure 2**), indicating that GABA decreases cell migration and invasion.

### Involvement of the GABA_A_ receptor in the inhibition of HCC cells

**Figure 3:**
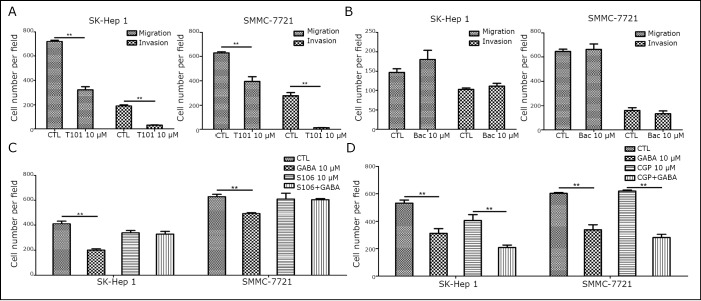
GABA-inhibited HCC migration and invasion via GABAA receptor. A, B: Migration and invasion assays were performed in SK-Hep1 and SMMC-7721 cells stimulated with 10 µM GABA_A_ or GABA_B_ receptor agonists for 24 h. Bac, baclofen. The results show the quantification of 5 randomly selected fields. C, D: Migration and invasion assays were performed in SK-Hep1 and SMMC-7721 cells stimulated with 10 µM GABA for 24 h with or without 2 h pre-incubation with GABA_A_ or GABA_B_ receptors antagonists. CGP, CGP 35348 hydrate. Data are representative of three independent experiments and are shown as means ±SEM (*, *P*<0.05, **, *P*<0.01, by Student’s *t*-test).

The effects of different agonists and antagonists were compared to identify a potential target receptor that is involved in the GABA-mediated inhibition of HCC cell migration and invasion. After administration of specific agonists for the GABA_A_ and GABA_B_ receptors in serum-free culture conditions, a marked decrease was observed in the number of migratory and invasive cells when the cells were incubated with GABA_A_ receptor agonists (T101), but not with GABA_B_ receptor agonists (Baclofen) (**Figure 3A** and **3B**). A decrease in the number of migratory and invasive cells was also found with muscimol, a selective GABA_A_ receptor agonist (data not shown). This finding was further supported when the cells were treated with S106 and CGP 35348 hydrate. When S106, a general GABA_A_ receptor antagonist, was added during incubation with GABA, GABA-mediated inhibition was suppressed. However, with CGP 35348 hydrate, a GABA_B_ receptor antagonist, the number of migratory and invasive cells decreased after incubation with GABA (**Figure 3C** and **3D**). Similar results were observed with B7561, a selective GABA_A_ receptor antagonist, and with 2-hydroxysaclofenwere, a GABA_B_ receptor antagonist (data not shown). In sum, the inhibition of GABA on the migration and the invasion of liver cancer cells is mediated by GABA_A_ receptors.

### Role of GABA in cytoskeletal reorganization

**Figure 4:**
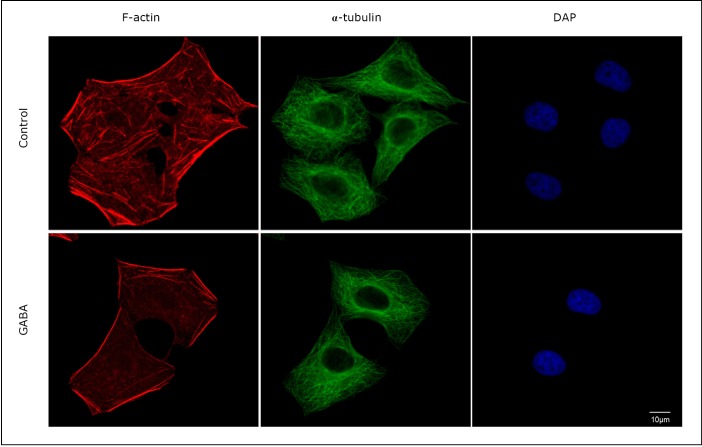
GABA-induced actin cytoskeleton reorganization. SMMC-7721 cells grown on an eight-well CultureSlide were fixed with 3.4% paraformaldehyde in PBS and then immunostained with Alexa Fluor^®^ 594 phalloidin (red), α-tubulin mouse monoclonal antibody (green), and DAPI (blue). Cytoskeletal changes occurred when the cells were stimulated with GABA. Scale bar=10 µm.

The actin cytoskeleton reportedly plays an important role in coordinating cell migration. Thus, the cytoskeletal changes were examined after the SMMC-7721 cells were stimulated with GABA. **Figure 4** shows that cytoskeletal changes occurred when the cells were stimulated with GABA. A comparison of the pretreated and control groups clearly demonstrated a decrease in the number of actin fibers in the cells stimulated with GABA. In addition, actin filaments were well organized only in GABA-treated cells. A type of F-actin stress fiber was observed in the control cells. Thus, these data verify that GABA regulates the formation of actin fibers.

### Evaluation of GABA in *in vivo* orthotopic nude mouse model

**Figure 5:**
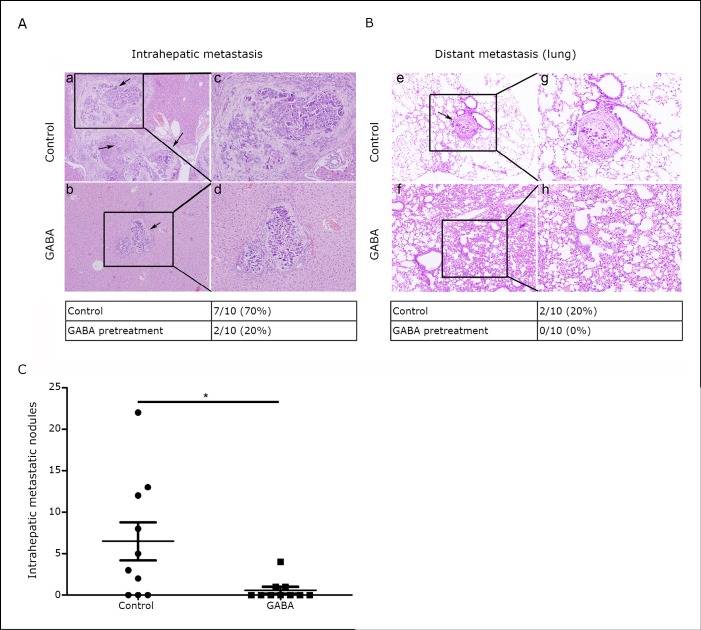
GABA-inhibited HCC cell invasion and metastasis *in vivo*. A, B: Hematoxylin-eosin-stained sections of intrahepatic metastatic nodules and distal metastatic nodules in the lung formed by SMMC-7721 cells with or without GABA pretreatment at the eighth week after intraperitoneal transplantation. Black arrows indicate the metastatic foci in the liver or the lung. Magnification: a, b, e, and f, × 100; c, d, g, and h, × 200. Tumor intrahepatic metastasis and distant lung metastasis were determined by histology. Metastasis frequency was then calculated. C: The numbers of metastatic nodules in each mouse liver were counted.

From our *in vitr*o experiments, the effects of GABA in an *in vivo* orthotopic model of liver cancer was subsequently tested. The HCC cell line SMMC-7721, which has relatively strong *in vitro* invasive properties, has been employed in *in vivo* metastasis assays in nude mice ^[^^29^^]^. Thus, the effect of GABA on the formation of primary and metastatic tumors was evaluated by injecting 5 × 10^5^ SMMC-7721 cells pretreated with 10 µM GABA or vehicle control into the liver of each mouse. Eight weeks later, a necropsy was performed to determine tumor growth and metastatic pattern. In the control group, 7 of 10 mice developed intrahepatic liver metastasis compared with only 2 of 10 mice in the GABA group (**Figure 5A**, *P*=0.021). Regarding the metastatic spread to the lungs, none of the mice in the GABA group developed distant lung metastases while 2 of 10 mice in the control group had lung metastases (**Figure 5B**, *P*=0.084). Interestingly, the number of metastatic nodules in the liver dramatically decreased in the GABA group when compared with the vector controls (**Figure 5C**, *P*=0.023).

## Discussion

Tumor cell migration is a prerequisite for invasion and metastasis, which account for more than 90% of cancer mortality. The signal substrates of the neuroendocrine systems are important in regulating cell migration. GABA, an inhibitory neurotransmitter in the adult mammalian nervous system, is also involved in modulating the proliferation, differentiation, and migration of several kinds of cells, including cancer cells. The functional regulatory roles of GABA in cancer cell growth and migration may differ in various types of cancer. In this study, the mRNA level of GABA receptor subunits is different between primary HCC tissues and adjacent NT liver tissues. However, GABA does not affect SK-Hep1 and SMMC-7721 cell proliferation, which may not conform to other studies due to the varied experimental conditions ^[^^24^^, ^^30^^]^. Cell migration and invasion are also suppressed in human liver cancer cells by the GABA treatment. GABA is known to exert its effects via the ionotropic GABA_A_ receptor and/or the metabotropic GABA_B_ receptor. Through the use of specific GABA receptor ligand agonists and antagonists, this study demonstrated that GABA inhibits human liver cancer cell migration and invasion through the GABA_A_ receptor.

Cytoskeletal alterations contribute to numerous features of aggressive tumors, such as increased cell motility, weakened adhesive contacts, and metastatic dissemination ^[^^31^^]^. In this study, GABA treatment decreases the number of actin stress fibers. Accumulated evidence highlights a process termed as the epithelial-mesenchymal transition, which results in numerous cytoskeletal alterations, such as expression of the intermediate filament vimentin, and facilitates cell invasion and metastasis ^[^^32^^, ^^33^^]^. This study also examined whether GABA can promote reverse transition or mesenchymal-epithelial transition. However, expressions of vimentin and N-cadherin, as well as β-catenin and ZO-1, do not vary between the GABA treated cells and the control cells (data not shown). Since the activation of GABA receptors stimulates PKA and PKC kinases, the activation of PKA and PKC/Ras/MAPK cascade is evaluated. However, the treatment of liver cancer cells with GABA does not result in changes in intracellular cAMP concentration, PKA kinase activity, or phosphorylation of CREB. In addition, pre-incubation with GABA fails to activate PKC kinase or promote the phosphorylation of JNK, p38, and ERK in liver cancer cells (data not shown). Further investigation is required to determine the downstream signals involved in the GABA-mediated inhibition of HCC cell migration and invasion.

As an inhibitory neurotransmitter, GABA is clinically used as a dietary supplement to reduce anxiety and to promote sleep. Previous studies focused on the potential use of GABA agonists or antagonists in direct anticancer therapies, as tested in both *in vitro* and *in vivo* chemopreventive experiments, due to the realization that GABA is important in regulating cancer cell migration ^[^^34^^, ^^35^^]^. Using an orthotopic mouse model, this study showed that pretreatment with GABA significantly inhibits intrahepatic liver metastasis and primary tumor formation. The control group has a higher rate of distant lung metastasis than the GABA group. However, a significant difference is not exhibited. Therefore, experiments that involve cells with stronger migration potential are necessary to confirm our observations.

In conclusion, the induced migration and invasion of liver cancer cells are suppressed by the neurotransmitter GABA *in vitro* and *in vivo* due to the induction of liver cancer cell cytoskeletal reorganization. In addition, the inhibitory effects of GABA are mediated by the ionotropic GABA_A_ receptor. Although further investigation is required on the role of GABA and its receptors in liver cancer metastasis, modulation of the GABAergic system, possibly combined with chemotherapy, may have potential therapeutic value in preventing cancer progression or metastasis, especially in treating cancer patients with metastasis.
